# Rap1 in the VMH regulates glucose homeostasis

**DOI:** 10.1172/jci.insight.142545

**Published:** 2021-06-08

**Authors:** Kentaro Kaneko, Hsiao-Yun Lin, Yukiko Fu, Pradip K. Saha, Ana B. De la Puente-Gomez, Yong Xu, Kousaku Ohinata, Peter Chen, Alexei Morozov, Makoto Fukuda

**Affiliations:** 1 Children’s Nutrition Research Center, Department of Pediatrics, Baylor College of Medicine, Houston, Texas, USA.; 2 Division of Food Science and Biotechnology, Graduate School of Agriculture, Kyoto University, Uji, Kyoto, Japan.; 3 Department of Molecular Medicine and Metabolism, Research Institute of Environmental Medicine, Nagoya University, Nagoya, Japan.; 4 Department of Medicine and; 5 Department of Molecular and Cellular Biology, Baylor College of Medicine, Houston, Texas, USA.; 6 Department of Medicine, Division of Pulmonary and Critical Care Medicine, Women’s Guild Lung Institute, Cedars-Sinai Medical Center, Los Angeles, California, USA.; 7 Unit on Behavioral Genetics, Laboratory of Molecular Pathophysiology, National Institute of Mental Health, NIH, Maryland, USA.; 8 Fralin Biomedical Research Institute at Virginia Tech Carilion, Roanoke, Virginia, USA.

**Keywords:** Metabolism, Neuroscience, G proteins, Glucose metabolism, Signal transduction

## Abstract

The hypothalamus is a critical regulator of glucose metabolism and is capable of correcting diabetes conditions independently of an effect on energy balance. The small GTPase Rap1 in the forebrain is implicated in high-fat diet–induced (HFD-induced) obesity and glucose imbalance. Here, we report that increasing Rap1 activity selectively in the medial hypothalamus elevated blood glucose without increasing the body weight of HFD-fed mice. In contrast, decreasing hypothalamic Rap1 activity protected mice from diet-induced hyperglycemia but did not prevent weight gain. The remarkable glycemic effect of Rap1 was reproduced when Rap1 was specifically deleted in steroidogenic factor-1–positive (SF-1–positive) neurons in the ventromedial hypothalamic nucleus (VMH) known to regulate glucose metabolism. While having no effect on body weight regardless of sex, diet, and age, *Rap1* deficiency in the VMH SF1 neurons markedly lowered blood glucose and insulin levels, improved glucose and insulin tolerance, and protected mice against HFD-induced neural leptin resistance and peripheral insulin resistance at the cellular and whole-body levels. Last, acute pharmacological inhibition of brain exchange protein directly activated by cAMP 2, a direct activator of Rap1, corrected glucose imbalance in obese mouse models. Our findings uncover the primary role of VMH Rap1 in glycemic control and implicate Rap1 signaling as a potential target for therapeutic intervention in diabetes.

## Graphical abstract

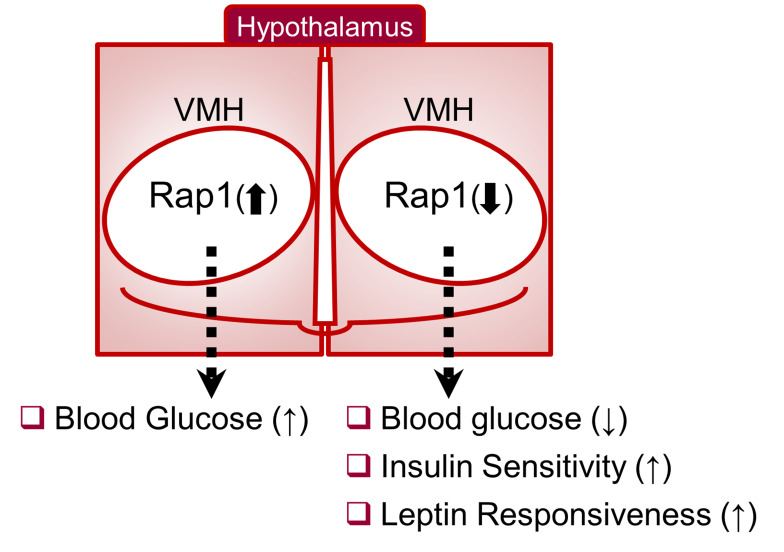

## Introduction

The brain has long been known as a key regulator of glucose metabolism (1–3). Recent studies have shown that the brain is clearly capable of correcting diabetic conditions (1, 2, 4–6). For example, direct infusion of leptin, insulin, and fibroblast growth factors into the brain exhibits a remarkable antidiabetic effect in animal models of diabetes (7–14). Clinical and preclinical data demonstrate that pharmacological activation of hypothalamic K_ATP_ channels or the serotonin 2C receptor improves glycemic control (15–17). In addition, deep brain stimulation was further shown to enhance peripheral insulin sensitivity in humans with diabetes (18). Thus, a growing body of evidence strongly suggests the brain as a promising yet unrealized therapeutic target for type 2 diabetes. To further materialize this concept, it is of great interest to identify potentially druggable molecular targets mediating the brain’s antidiabetic effects.

One of the critical hypothalamic sites mediating glycemic control is the ventromedial nucleus of the hypothalamus (VMH). The VMH has been recognized as a hypothalamic nucleus that possesses glucose-sensing neurons (19, 20) and regulates glucose metabolism of peripheral tissues (21, 22). Recent genetic and pharmacological studies have demonstrated that multiple hormonal and neural signals regulate VMH neurons to alter glucose balance (23–31). Further evidence supporting the role of VMH in glucose metabolism stems from optogenetic, electromagnetic, and chemogenetic studies, demonstrating that manipulations of VMH neural activity influence blood glucose levels, glucose tolerance, and peripheral insulin sensitivity (27, 32–38). Consequently, VMH neurons are thought to be a crucial mediator of the neural glucoregulatory mechanism. However, signaling mechanisms within VMH neurons that mediate whole-body glycemic control remain elusive.

Rap1 is a monomeric small GTPase belonging to the Ras family (39). Rap1, which is encoded by *Rap1a* and *Rap1b*, is ubiquitously expressed throughout the body. At the cellular level, Rap1 mediates various cellular functions, such as proliferation, differentiation, adhesion, and motility (40). In the central nervous system (CNS), Rap1 is widely expressed in broad areas of the CNS, including hypothalamic nuclei known to control energy and glucose homeostasis (41, 42). Rap1 in the CNS is activated in response to acute and chronic high-fat diet (HFD) feeding (42, 43). Furthermore, activation of Rap1 diminishes cellular actions of leptin, a crucial hormonal mediator that maintains normal body weight in vitro and in vivo, thereby contributing to adiposity (43, 44). In addition, genetic deletion of *Rap1* in the forebrain protects mice from HFD-induced metabolic disturbances, such as neural leptin resistance, obesity, and glucose imbalance (42). Consistently, mice with global knockout of Exchange protein directly activated by cAMP 1, a GTP/GDP exchange factor for Rap1 (an upstream activator of Rap1), are also protected from diet-induced obesity and insulin resistance (45). As such, Rap1 signaling in the CNS has emerged as a crucial mediator for the effects of HFD feeding, including the development of leptin resistance, obesity, and glucose imbalance. However, the exact CNS sites and the specific neural populations where Rap1 mediates overnutrition-associated disorders remain to be determined. Here, we defined the physiologic function of Rap1 expressed by the medial hypothalamus, a key CNS site for the control of energy and glucose metabolism, by employing a combination of gain-of-function and loss-of-function genetics, pharmacology, and glucose clamp studies.

## Results

### Hypothalamic Rap1 controls blood glucose but not body weight in overnutrition conditions.

**Figure 1:**
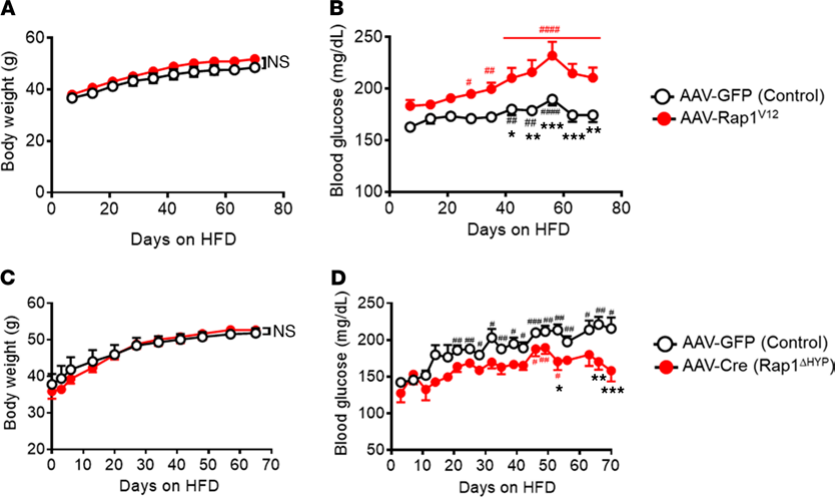
Rap1 mediates hyperglycemia in the hypothalamus. (**A** and **B**) Increased hypothalamic Rap1 activity aggravates hyperglycemia under HFD conditions. Body weight (**A**) and blood glucose (**B**) of Rap1^V12^ mice are shown. Six weeks after AAV-Rap1^V12^ injection, mice were subjected to HFD feeding (*n* = 12/group). (**C** and **D**) Hypothalamic deletion of *Rap1a* and *Rap1b* prevents HFD-induced hyperglycemia. Shown are the body weight (**C**) and blood glucose (**D**) of Rap1^ΔHYP^ mice. One week after AAV2-Cre injection into the medial hypothalamus of the *Rap1a* and *Rap1b* double-floxed mice, HFD feeding started (*n* = 12–13/group). Values are presented as the mean ± SEM. ^#^*P* < 0.05, ^##^*P* < 0.01, and ^###^*P* < 0.001 compared with each group on day 0, by 1-way ANOVA followed by Dunnett’s multiple comparisons test, and *P < 0.05, **P < 0.01, and ***P < 0.001 compared with the control group, by repeated measures 2-way ANOVA with Bonferroni’s multiple comparisons test.

While Rap1 activity in the hypothalamus is increased in response to acute and chronic HFD feeding (42, 43), it remains unclear whether increased activity of hypothalamic Rap1 plays a role in HFD-induced body weight gain and glucose imbalance. To directly test this, we increased hypothalamic Rap1 activity by bilaterally injecting adeno-associated virus (AAV) expressing a constitutively active GTP-locked human *Rap1a* variant (46) (AAV-Rap1^V12^) into the medial hypothalamic area aimed at the VMH. As a control, AAV expressing GFP alone was injected. After the injections of AAVs, the mice were challenged with an HFD to induce diet-induced obesity and hyperglycemia. AAV-mediated ectopic expression of human Rap1^V12^ was confirmed in the medial hypothalamic area including the VMH by using quantitative PCR (qPCR) (Supplemental Figure 1A; supplemental material available online with this article; https://doi.org/10.1172/jci.insight.142545DS1), and GFP fluorescence was found in the VMH (Supplemental Figure 1B), suggesting the VMH as a primary site of hypothalamic expression of Rap1^V12^. Hypothalamic expression of Rap1^V12^ resulted in a modest yet significant increase in total Rap1 activity (a 1.6-fold increase, Supplemental Figure 1C). Prior to the onset of HFD feeding, there were no differences in body weight and blood glucose levels between AAV-Rap1^V12^ and their control mice (Supplemental Figure 1, D and E). During HFD feeding, both AAV-Rap1^V12^ and control mice were similar in body weight (Figure 1A). However, blood glucose was markedly elevated in AAV-Rap1^V12^ mice compared with control animals (Figure 1B). Rap1-induced hyperglycemia was not observed under normal chow–fed conditions, as demonstrated by the results that forced activation of hypothalamic Rap1 had no impact on body weight and blood glucose in lean animals (Supplemental Figure 1, F and G). These data collectively suggest that increased Rap1 activity in the hypothalamus sufficiently aggravates diet-induced hyperglycemia without an effect on body weight.

The glycemic effect might be due to the pharmacological effects of overexpression of a constitutively active form of Rap1 in the medial hypothalamus, and we next sought to investigate the physiological relevance of hypothalamic Rap1. To do this, we decreased Rap1 within the hypothalamus using bilateral injection of AAV expressing Cre recombinase into the medial hypothalamus aimed at the VMH of *Rap1a* and *Rap1b* double-floxed mice (47) (Rap1^ΔHYP^). For the control, we injected GFP-expressing AAVs into the medial hypothalamus (control). GFP fluorescence was found in the VMH and to a lesser extent in the dorsomedial hypothalamus but not the arcuate nucleus (ARC) (Supplemental Figure 1H). Consistently, *Rap1a* and *Rap1b* mRNAs were significantly reduced in the VMH of AAV-Cre–injected mice (Supplemental Figure 1I). While blood glucose levels of control mice were significantly elevated over the course of HFD feeding, Rap1^ΔHYP^ mice did not show increased blood glucose in response to HFD feeding (Figure 1D), suggesting that genetic deletion of hypothalamic Rap1 prevents HFD-induced hyperglycemia. Interestingly, body weight was not altered by hypothalamic deletion of Rap1 (Figure 1C). Thus, AAV-mediated deletion of *Rap1* genes restricted to the hypothalamus protects mice against HFD-induced mild hyperglycemia independent of body weight. These loss-of-function and gain-of-function data collectively highlight the primary role of hypothalamic Rap1 in mediating hyperglycemia during HFD conditions.

### Production and validation of steroidogenic factor-1–specific Rap1-deficient mice.

**Figure 2:**
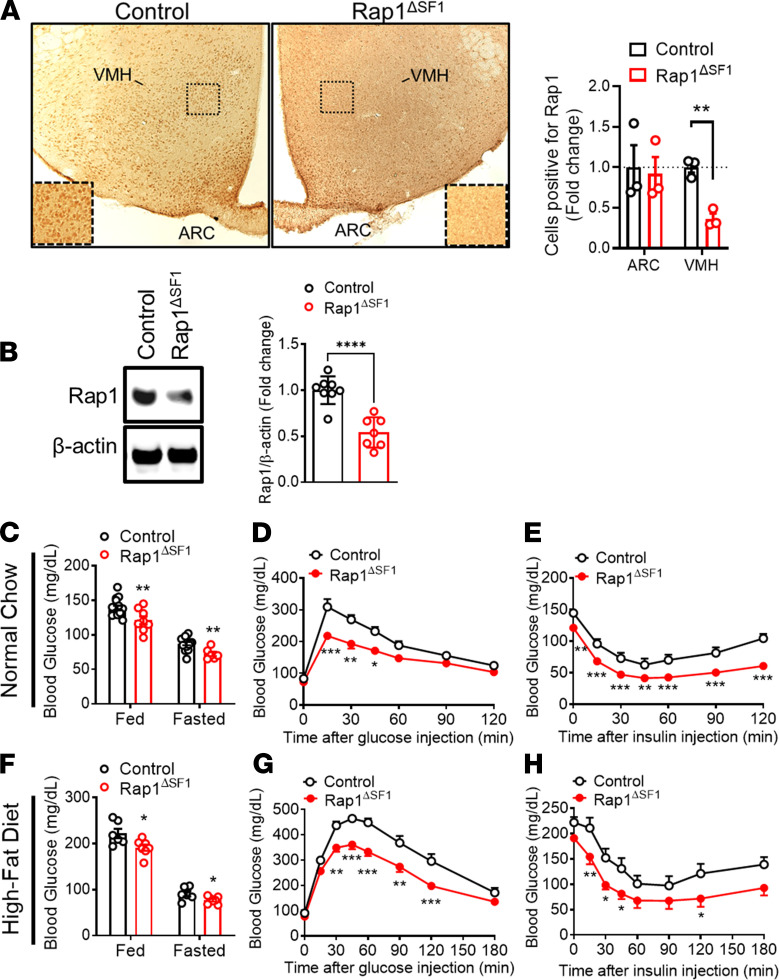
Improved glucose homeostasis in Rap1^ΔSF1^ mice. (**A**) Representative images (original magnification, ×100; inset, original magnification, ×180) and quantification of Rap1 immunoreactivity in the hypothalamus of Rap1^ΔSF1^ mice and control mice. The number of Rap1-positive cells in the ARC and the VMH was counted and is represented as a fold change relative to control (*n* = 3). (**B**) Representative Western blots and densitometric quantification of VMH Rap1 expression normalized to β-actin. (*n* = 7–8/group). See complete unedited blots in the supplemental material. (**C**–**E**) Glucose profiles of Rap1^ΔSF1^ or control mice under normal chow conditions. Blood glucose (**C**, *n* = 6–15), glucose tolerance testing (**D**, *n* = 8), and insulin tolerance testing (**E**, *n* = 8) were measured. (**F**–**H**) Glucose profiles of HFD-fed Rap1^ΔSF1^ or control mice (14 weeks of HFD feeding, *n* = 6–7). Glucose (**F**), glucose tolerance testing (**G**), and insulin tolerance testing (**H**). *P < 0.05, **P < 0.01, ***P < 0.001, and ****P < 0.0001 for 2-tailed *t* tests (**A**–**C** and **F**) or 2-way ANOVA followed by Bonferroni’s multiple comparisons tests (**D**, **E**, **G**, and **H**). All error bars are mean ± SEM.

While Rap1 in the forebrain (42) and in the hypothalamus (Figure 1 in this study) plays a critical role in glycemic regulation, the site of the effect remains to be established. We assume that VMH neurons mediate Rap1-dependent glycemic control on the basis of the following observations: (a) the VMH is a well-established hypothalamic nucleus including multiple distinct neural populations that influence whole-body glucose metabolism (20, 33, 48, 49); (b) Rap1 is produced in the VMH (ref. 42 and Figure 2A); (c) in Rap1^ΔCNS^ mice that have improved glucose balance, Rap1 is depleted in the VMH (42); and (d) AAV-mediated manipulation of Rap1 occurs mostly in the VMH but not in the ARC (Supplemental Figure 1, B and H). Thus, we next sought to determine whether Rap1 participates in VMH-mediated glycemic control. To this end, we generated mice lacking Rap1 specifically in VMH neurons, referred to herein as Rap1^ΔSF1^ mice, by breeding *Rap1a* and *Rap1b* double-floxed mice (47) to the steroidogenic factor-1 (SF1) *Cre* line that expresses *Cre* recombinase only in SF-1–positive neurons in the brain (23). VMH SF1 neurons are a critical subset of VMH neurons for the control of leptin actions, energy, and glucose homeostasis. We first confirmed Cre-mediated excision of the floxed *Rap1* alleles in the hypothalamus including the VMH (Supplemental Figure 2A). Immunohistochemical analyses for endogenous Rap1 exhibited selective depletion of Rap1 protein in the VMH but not in the adjacent ARC (Figure 2A), which was confirmed by Western blot analysis of the Rap1 protein (Figure 2B and Supplemental Figure 3A). We further confirmed that Rap1 was markedly reduced in SF1-positive cells in the VMH (Supplemental Figure 2B). In addition, the vast majority of SF1 cells were concomitantly labeled with NeuN, a marker of postmitotic neurons (50) (Supplemental Figure 2C), suggesting neuron-specific deletion of Rap1 in the VMH. Altogether, our results demonstrate that Rap1 is produced in the VMH and that its deletion is restricted to SF1 neurons in Rap1^ΔSF1^ mice.

### Improved glucose balance and peripheral insulin sensitivity in Rap1ΔSF1 mice.

Using Rap1^ΔSF1^ mice, we directly examined whether Rap1 in the VMH has a role in systemic glucose balance. Under normal chow condition, Rap1^ΔSF1^ mice exhibited a significant decrease in blood glucose in both the fed and fasted states (Figure 2C) and lower serum insulin levels but not serum glucagon (Supplemental Figure 4). In agreement with lower glycemia, Rap1^ΔSF1^ animals had markedly improved glucose and insulin tolerance compared with weight- and age-matched littermate controls (Figure 2, D and E). We further examined the effect of SF1 cell–specific Rap1 deletion on HFD-induced diabetes-like conditions. HFD-fed Rap1^ΔSF1^ mice showed significantly reduced blood glucose levels (Figure 2F), improved glucose tolerance (Figure 2G), and enhanced insulin sensitivity (Figure 2H) compared with control littermates under HFD conditions. Improved glucose balance was similarly observed in HFD-fed female Rap1^ΔSF1^ mice (Supplemental Figure 5, B–D). Thus, these data suggest that SF1 cell–specific Rap1 deficiency protects mice from HFD-induced insulin resistance, hyperglycemia, and impaired glucose tolerance.

**Figure 3:**
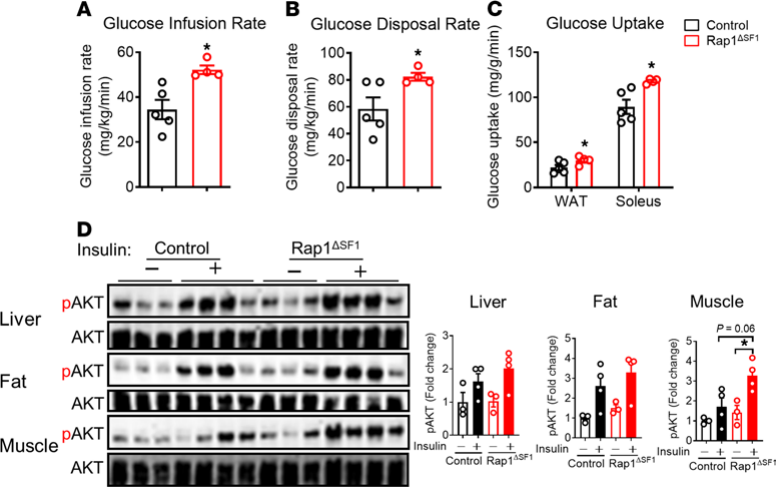
Improved insulin sensitivity in Rap1^ΔSF1^ mice. (**A**–**C**) Hyperinsulinemic-euglycemic clamp studies in Rap1^ΔSF1^ and littermate control mice fed an HFD for 18 weeks (*n* = 4–5). Shown are the GIR (**A**), peripheral glucose disposal rate (**B**), and 2-deoxy-d-glucose (2DG) uptake (**C**). (**D**) Western blot (left) and quantification (right) of AKT (Thr308) and glycogen synthase kinase-3β (Ser9) phosphorylation in liver, fat, and muscle at 10 minutes after a bolus injection of insulin (1 U/kg, i.p.) or saline into Rap1^ΔSF1^ or control mice fed an HFD for 35 weeks. See complete unedited blots in the supplemental material. *P < 0.05 for 2-tailed *t* tests (**A**–**C**) or 2-way ANOVA followed by Bonferroni’s multiple comparisons tests (**D**). All error bars are mean ± SEM.

To further characterize the mechanisms underlying the improved glucose and insulin homeostasis in Rap1^ΔSF1^ mice, hyperinsulinemic-euglycemic clamp studies were performed in HFD-fed Rap1^ΔSF1^ and littermate control mice. Compared with control animals, Rap1^ΔSF1^ mice displayed a significantly higher glucose infusion rate (GIR) to maintain euglycemia (Figure 3A), indicating that Rap1 in the VMH neurons controls whole-body insulin actions. This increase in GIR in Rap1^ΔSF1^ mice seems to be due to an increase in insulin-induced peripheral glucose disposal (Figure 3B). Consistently, 2-deoxy-d-glucose (2DG) uptake in muscles and adipose tissue was significantly increased in SF1 neuron–specific Rap1-deficient mice (Figure 3C). As shown in Figure 3D, cellular insulin sensitivity was also enhanced in the muscle of Rap1^ΔSF1^ mice compared with littermate control mice, which is clearly demonstrated by enhanced insulin-induced phosphorylation of AKT, a critical mediator of cellular insulin signaling in Rap1^ΔSF1^ mice.

In addition to Rap1 deletion in the VMH in the CNS, Rap1 was ablated in adrenal glands (Supplemental Figure 2A and Supplemental Figure 3B) as expected. Loss of Rap1 in the adrenal glands is unlikely to account for the glucose phenotype of Rap1^ΔSF1^ mice. First, basal and stress-induced corticosterone levels were similar between control and Rap1^ΔSF1^ mice (Supplemental Figure 3D), indicating preserved adrenal function. Furthermore, the adrenal glands of Rap1^ΔSF1^ mice were histologically indistinguishable from those of control mice (Supplemental Figure 3E). More importantly, AAV-mediated deletion of Rap1 only in the VMH and its surrounding areas sufficiently induced the glucose phenotype without affecting peripheral Rap1 (Figure 1). These data indicate that the observed phenotype in these mice is highly likely to be due to the absence of Rap1 in VMH SF1 neurons.

### Energy balance and leptin responsiveness in Rap1ΔSF1 mice.

**Figure 4:**
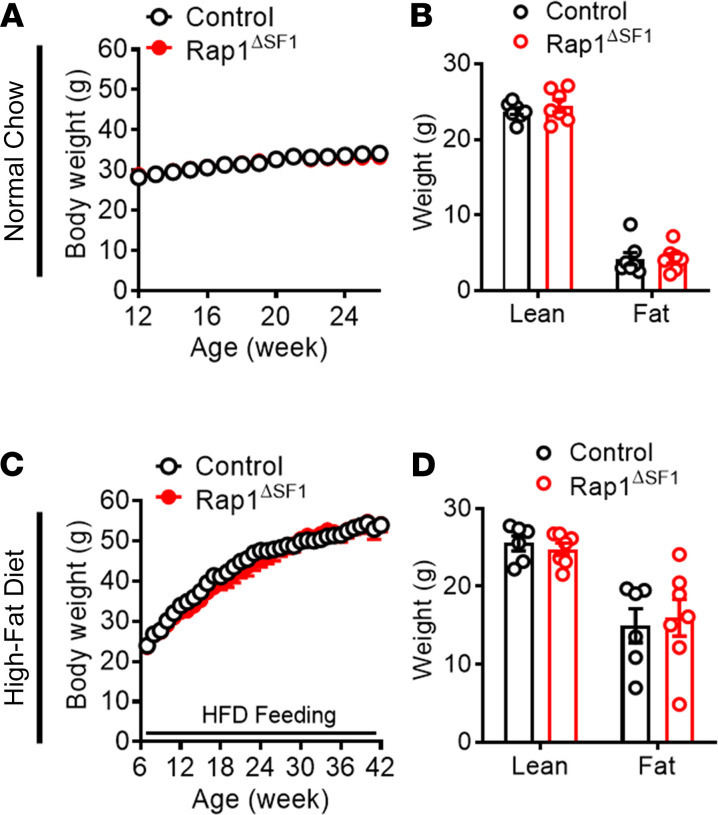
Body weight and adiposity of Rap1^ΔSF1^ mice. Shown are the weekly body weight (**A**) and body composition (**B**) in normal chow–fed male Rap1^ΔSF1^ mice or control mice (*n* = 9–14). Additionally, shown are the weekly body weight (*n* = 10–15) (**C**) and body composition (*n* = 6–7) (**D**) under HFD conditions. The HFD was initiated at 7 weeks of age, and body composition was measured after 17 weeks of HFD feeding. All error bars are mean ± SEM.

**Figure 5:**
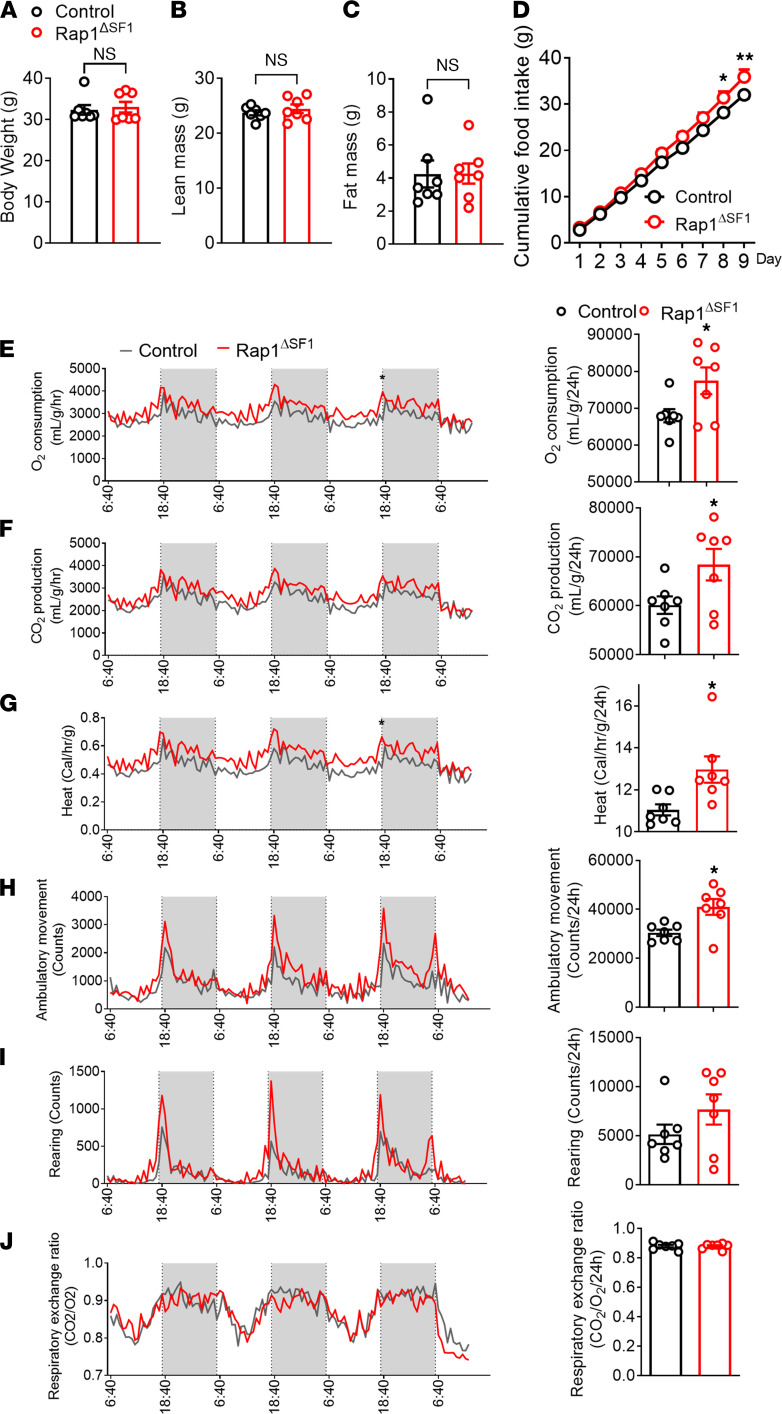
Food intake and energy expenditure of Rap1^ΔSF1^ mice. Metabolic profiles of male Rap1^ΔSF1^ mice or control mice (*n* = 7 per group) with respect to body weight (**A**), lean mass (**B**), fat mass (**C**), food intake (**D**), O_2_ consumption (**E**), CO_2_ production (**F**), heat production (**G**), ambulatory activity (**H**), rearing (**I**), and respiratory exchange ratio (**J**). *P < 0.05 for 2-tailed *t* tests (**A**–**C** and **E**–**J**) or 2-way ANOVA followed by Bonferroni’s multiple comparisons tests (**D**). All error bars are mean ± SEM.

Because forebrain-specific *Rap1*-knockout mice display a lean phenotype (42), it is possible that the remarkable glycemic effects we observed in Rap1^ΔSF1^ mice might be due to the potential confounding effects of *Rap1* deficiency on body weight or adiposity. We thus explored the effect of Rap1 in VMH SF1 neurons on energy balance by measuring the body weight and adiposity of Rap1^ΔSF1^ and their littermate control mice. We found no differences in body weight and adiposity between the 2 groups irrespective of age, diet, or sex (Figure 4, A–D; Figure 5, A–C; and Supplemental Figure 5A). Despite an apparent lack of effect on body weight, Rap1^ΔSF1^ mice exhibited increased food intake (Figure 5D). Concomitantly, parameters pertaining to energy expenditure were significantly increased in the mice with SF1 cell–specific Rap1 deletion compared with littermate controls by dual-energy x-ray absorptiometry analysis (Figure 5, E–J). These data suggest that Rap1 deletion in SF1 cells has no impact on body weight, which is likely to be accounted for by a simultaneous increase in both food intake and energy expenditure by Rap1 deficiency in SF1 neurons.

**Figure 6:**
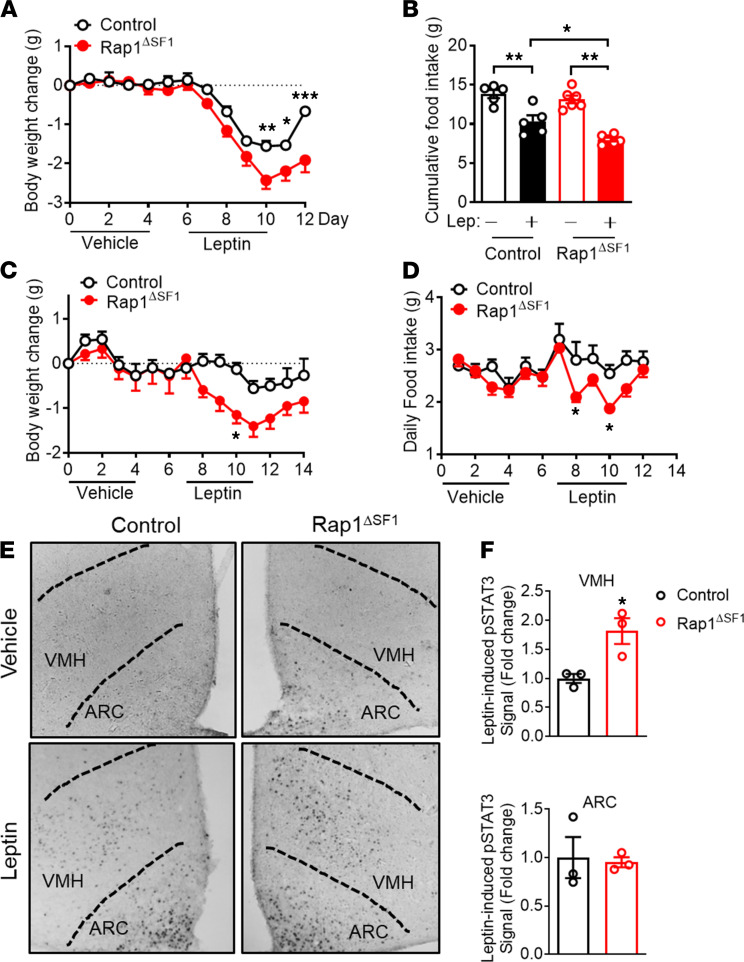
Leptin responsiveness is increased in Rap1^ΔSF1^ mice. (**A** and **B**) Leptin (3 mg/kg, twice a day, i.p.) or vehicle was administered to normal chow–fed lean Rap1^ΔSF1^ or control mice (*n* = 5–6). Shown are the body weight (**A**) and cumulative food intake (**B**). (**C** and **D**) HFD-fed Rap1^ΔSF1^ or control mice (15 weeks of HFD) were injected with leptin (3 mg/kg, twice per day, i.p.) or vehicle (*n* = 6–7). Body weight (**C**) and food intake (**D**) were measured every day. (**E** and **F**) Leptin (3 mg/kg, i.p.) was administered to the indicated mice (*n* = 3 per group). (**E**) Representative immunohistochemistry images for phosphorylated STAT3, original magnification, ×100. (**F**) Quantification of immunohistochemistry. Age- and body weight–matched cohorts were used (**A**–**F**). *P < 0.05, **P < 0.01, and ***P < 0.001 for 2-tailed *t* tests (**F**), 1-way ANOVA followed by Tukey’s multiple comparison test (**B**) or 2-way ANOVA followed by Bonferroni’s multiple comparisons test (**A**, **C**, and **D**). All error bars are mean ± SEM.

Since Rap1 is known to inhibit the cellular action of leptin (42, 44, 45), a critical hormone that maintains normal body weight and normoglycemia, we examined the effect of VMH Rap1 on leptin responsiveness. By assessing cellular and anorectic responses to exogenously administered leptin (3 mg/kg, i.p., twice a day), we found that Rap1^ΔSF1^ mice showed greater weight loss in response to leptin compared with the control animals (Figure 6A). In addition, leptin was more effective at suppressing food intake in Rap1^ΔSF1^ mice than in their controls (Figure 6B), suggesting the enhanced leptin responsiveness in Rap1^ΔSF1^ mice. Under HFD conditions, the response to exogenous leptin was diminished in the control mice (Figure 6, C and D), as previously shown (51). Rap1^ΔSF1^ mice retained their ability to respond to exogenous leptin in terms of leptin-induced suppression of food intake and reduction in body weight (Figure 6, C and D). This finding concurs with the increased leptin-dependent phosphorylation of STAT3, a critical mediator for leptin’s metabolic effects, in the VMH but not in the ARC of Rap1^ΔSF1^ mice (Figure 6, E and F). These results suggest that despite having no effect on body weight/adiposity, Rap1 deficiency sensitizes VMH SF1 neurons to exogenous leptin action in vivo.

### Acute pharmacological inhibition of brain exchange protein directly activated by cAMP 2 improves glucose balance independent of leptin.

**Figure 7:**
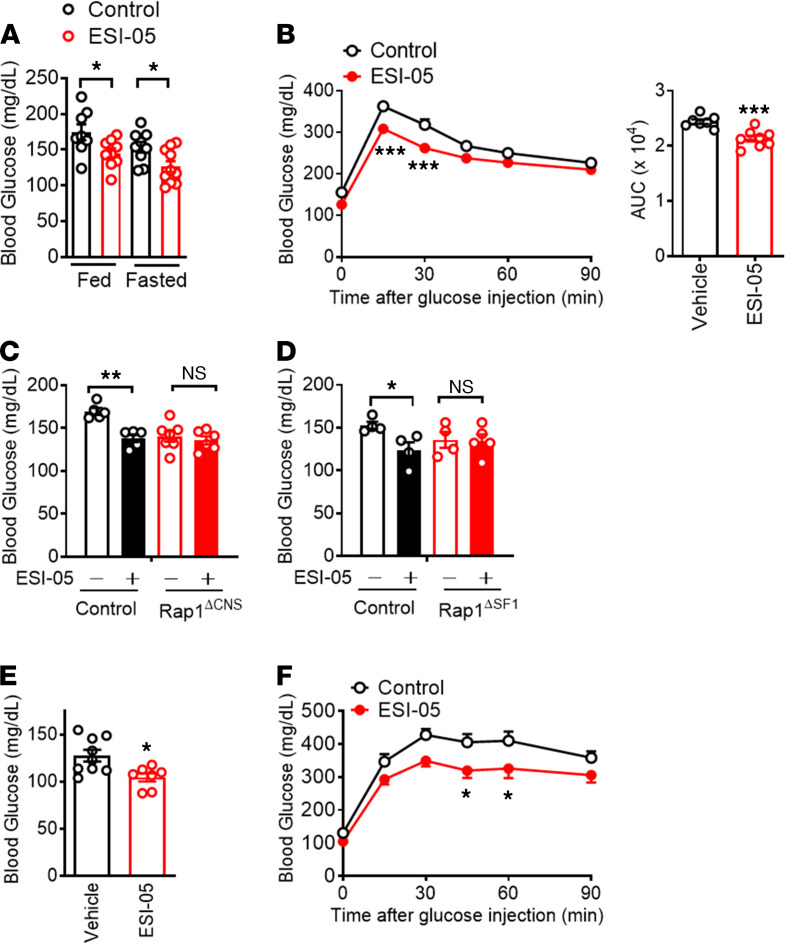
ESI-05 improves glucose homeostasis independent of leptin action. (**A** and **B**) Centrally administered ESI-05 (0.2 nmol/mouse, twice a day) lowered blood glucose (**A**) and improved glucose tolerance (**B**) in HFD-fed C57BL/6J mice (21 weeks of HFD). Age- and body weight–matched cohorts were used (*n* = 7–10). (**C** and **D**) The glucose-lowering effect of ESI-05 (0.2 nmol, twice a day) was not observed in brain-specific *Rap1*-deficient mice (Rap1^ΔCNS^) fed an HFD for 5 weeks (*n* = 5–7) or in 6-week HFD-fed Rap1^ΔSF1^ mice (*n* = 4–5). (**E** and **F**) Effect of ESI-05 (i.c.v., 0.2 nmol/mouse, twice a day) on blood glucose and glucose tolerance in *ob/ob* mice. Shown are the fed blood glucose (**E**) and glucose tolerance (**F**). *P < 0.05, **P < 0.01, and ***P < 0.001 for 2-tailed *t* tests (**A**–**E**) or 2-way ANOVA followed by Bonferroni’s multiple comparisons test (**B** and **F**). All error bars are mean ± SEM.

We sought to assess the potential translational relevance of CNS Rap1 inhibition. To test this, we used ESI-05, a well-established selective inhibitor of exchange protein directly activated by cAMP 2 (EPAC2) (52, 53). EPAC2 is a GTP/GDP exchange factor for Rap1 that serves as a direct activator for Rap1 and is highly enriched in the brain (54). We have previously demonstrated that ESI-05 successfully inhibited endogenous Rap1 activity in the hypothalamus when ESI-05 was centrally infused at a dose of 0.2 nmol (42, 55). The same dose of ESI-05 was directly infused into the brains of HFD-fed C57BL/6 mice, and we assessed the effect of ESI-05 on glucose metabolism. Centrally administered ESI-05 alleviated mild hyperglycemia in HFD-fed mice, as demonstrated by the lowered fed and fasted blood glucose levels (Figure 7A) and improved glucose tolerance (Figure 7B) in the HFD-fed mice treated with i.c.v. ESI-05 compared with levels in the vehicle-injected control HFD-fed mice. We confirmed the Rap1 dependency of ESI-05’s glycemic effect by demonstrating that the glucose-lowering effect of ESI-05 was not observed in Rap1^ΔCNS^ mice that lacked both *Rap1a* and *Rap1b* in the forebrain including the hypothalamus (Figure 7C). We further found that ESI-05–induced glucose lowering was completely abolished in Rap1^ΔSF1^ mice (Figure 7D), suggesting that Rap1 in VMH SF1 neurons mediated the glucose-reducing effect of ESI-05. Collectively, the acute pharmacological inhibition of Rap1 signaling improved HFD-induced disordered glucose balance, which further supports the genetic evidence described above.

### The glucose lowering of ESI-05 is independent of leptin action.

Rap1 deficiency in the forebrain (42) or in the VMH (this study) simultaneously enhances leptin responsiveness and improves glucose balance. As leptin is known to improve glucose balance, we clarified the potential role of leptin in the glucose-lowering effect of ESI-05. Centrally administered ESI-05 significantly lowered blood glucose levels (Figure 7E) and improved glucose tolerance (Figure 7F) in leptin-deficient obese mice (*ob/ob* mice), which is comparable to the effect in leptin-intact dietary obese mice (Figure 7, A and B), suggesting that brain Rap1-mediated glycemic regulation occurs without leptin. Collectively, our data clearly demonstrate that acute inhibition of Rap1 signaling in the brain improves glucose balance by a leptin-independent mechanism.

## Discussion

The CNS has emerged as an attractive target for diabetes intervention (1–6), although such an approach has not yet fully materialized. Thus, it is of paramount interest to identify druggable targets within the CNS glucoregulatory mechanism that would potentially create a novel therapeutic intervention to mitigate diabetic conditions. Our findings reveal a molecular pathway in the hypothalamus that mediates whole-body glucose balance, as we demonstrate that hypothalamic Rap1 is sufficient to produce alterations in whole-body glucose balance. Specifically, activation of Rap1 in the hypothalamus exaggerated hyperglycemia in diet-induced obesity. In contrast, hypothalamic loss of Rap1 decreased hyperglycemia in dietary obesity. The glycemic phenotype was observed without altered body weight, suggesting the primary role of Rap1 in glucoregulatory function. In addition, we provide a proof of concept for the potential of targeting Rap1 signaling within the CNS to improve glucose imbalance and induce antidiabetic effects. Our data collectively suggest that hypothalamic Rap1 is a molecular pathway for the control of glucose metabolism and mediates HFD-induced glucose imbalance, thereby making it a potential target for therapeutics.

The remarkable glycemic effect of hypothalamic Rap1 could be related to its action within the VMH neurons. The VMH is classically known as a critical brain site for the control of glucose metabolism (21, 33, 48, 49, 56). Modulation of neural activity of the VMH neurons, in particular, SF1-positive neurons, has been shown to influence glucose metabolism of peripheral tissues. Indeed, optogenetic activation of VMH SF1 neurons increased blood glucose by inducing counterregulatory responses (33, 38), whereas chemogenetic activation of VMH SF1 neurons enhanced peripheral insulin sensitivity and increased glucose disposal (34). In line with these previous studies, our data show that genetic ablation of Rap1 from SF1 neurons significantly decreased blood glucose, remarkably reduced serum insulin levels, and robustly improved glucose and insulin tolerance. As Rap1 expression remains intact in the pancreas of Rap1^ΔSF1^ mice (Supplemental Figure 2A and Supplemental Figure 3C), a marked reduction in insulin level is likely due to the central effect of Rap1 deficiency. Furthermore, Rap1^ΔSF1^ mice had an increased glucose infusion rate under the insulin clamp condition, suggesting enhanced whole-body insulin sensitivity. Consistently, cellular insulin signaling in skeletal muscle was significantly enhanced by Rap1 deficiency in VMH neurons. In addition, enhanced glucose uptake was further observed in the skeletal muscle and fat of Rap1^ΔSF1^ mice. Our findings, taken together with the data demonstrating that Rap1^ΔSF1^ mice had no significant impact on serum levels of hormones that promote glucose mobilization, Rap1 deficiency in the VMH is likely to improve glucose balance by promoting glucose disposal.

A single dose of insulin caused a greater fall in blood glucose in Rap1^ΔSF1^ mice (Figure 2, E and H; and Supplemental Figure 5D). Because insulin-induced hypoglycemia is usually limited by the counter-regulatory mechanism that is initiated in part by the brain including the VMH neurons, our data may indicate impaired counter-regulation to the insulin stimulus. Given that the VMH is the critical site for the counter-regulatory mechanism (38, 57) and that VMH Rap1 has emerged as a key regulator of glucose metabolism, it is possible that Rap1 in the VMH has a role in the counter-regulatory process. In addition, we found that i.c.v. glucose-induced c-Fos induction was significantly attenuated in the VMH, but not in the ARC, of mice receiving i.c.v. ESI-05 (Supplemental Figure 6). These results may indicate potential roles of VMH Rap1 signaling in glucose-sensing mechanisms and are worth future investigations. Our findings collectively highlight the importance of Rap1 in mediating VMH-dependent glycemic regulation.

While the VMH is known to play a major role in glycemic control, this nucleus also mediates other metabolic functions, including the regulation of energy balance (i.e., food intake, energy expenditure, adiposity, and body weight) (23, 28, 30, 31, 34, 35, 38). We examined the metabolic phenotypes of Rap1 deficiency in the VMH to determine the potential specificity of Rap1 signaling for improving glucose balance and found that VMH Rap1 was not involved in determining body weight irrespective of diet, sex, and age, although it influenced both arms of energy balance by increasing food intake and energy expenditure (O_2_ consumption, CO_2_ production, heat production, and physical activities), thereby offsetting the effect on body weight. Rather, VMH Rap1 deletion decreased blood glucose and enhanced cellular insulin signaling in peripheral tissues. Furthermore, Rap1^ΔSF1^ mice showed increased peripheral glucose utilization in HFD-induced obesity, suggesting the specific role of VMH Rap1 in glucoregulatory functions. Interestingly, our previous study demonstrated that mice with Rap1 deficiency in the broader area of the brain (using a Cre line expressing CaMKII-driven Cre recombinase with specific activity in the forebrain including multiple hypothalamic nuclei) exhibited markedly improved leptin responsiveness, reduced body weight and adiposity, and decreased food intake (42), all of which suggest the role of forebrain Rap1 in participating in energy balance. Compared with the prior study (42), our findings define the physiological functions of Rap1 in VMH SF1 neurons relative to the broader forebrain areas, as we propose that VMH Rap1 is a molecular pathway capable of selectively modulating glucose metabolism without having an effect on body weight.

Supporting the specific role of Rap1 in the VMH for glycemic regulation, signaling molecules that modulate and mediate Epac/Rap1 signaling also selectively exhibit their glucoregulatory role within VMH SF1 neurons. The heterotrimeric G protein Gs (Gsα) leads to activation of Epac/Rap1 signaling by promoting cAMP production (58). Interestingly, mice with Gsα deficiency in VMH SF1 neurons display phenotypes akin to those of VMH Rap1-knockout mice: enhanced leptin responsiveness and improved glucose balance without altering body weight (29). Similarly, VMH SF1 neuron–specific deletion of SOCS3, a major downstream mediator of Epac/Rap1 signaling, also exhibits the same phenotypes, such as increased leptin responsiveness and markedly improved glucose homeostasis in the absence of an effect on body weight (24). These striking similarities in the phenotypes and the fact that Rap1 is biochemically linked to these signaling molecules collectively suggest the critical role of the Rap1 pathway as an intracellular signaling modality in the VMH neurons capable of determining whole-body glucose balance.

Rap1^ΔSF1^ mice showed markedly enhanced cellular and anorectic responses to leptin, suggesting that Rap1 deficiency sensitizes leptin action in VMH SF1 neurons in vivo. Because leptin increases peripheral glucose uptake and corrects hyperglycemia when directly infused into the VMH (59, 60), it is possible that the effects observed in Rap1^ΔSF1^ mice are due to enhanced sensitivity of leptin. Although possible, this seems unlikely given that a similar glycemic effect was produced in the absence of leptin. Furthermore, despite improved leptin responsiveness, Rap1 deficiency in the VMH did not have an effect on body weight. This is somewhat surprising, considering that leptin is a major determinant of body weight. Nevertheless, while the precise role of increased leptin action of Rap1^ΔSF1^ mice is unknown, future studies are warranted to investigate a specific role of leptin in mediating the metabolic phenotypes of Rap1^ΔSF1^ mice.

Last, one important implication arising from this study is that targeting the CNS Rap1 pathway might have the potential to yield antidiabetic effects because our pharmacological study demonstrates that ESI-05 significantly decreased fed and fasted blood glucose levels and improved glucose tolerance in HFD-induced obese mice (Figure 7). Identification of existing and novel chemical compounds that can modulate hypothalamic Rap1 activity may offer a novel therapeutic opportunity to improve type 2 diabetes.

In summary, our results offer genetic and pharmacological evidence suggesting that hypothalamic Rap1 signaling, especially in the VMH, is a pivotal pathway for the control of glucose homeostasis. The findings also unveiled an antidiabetic effect of Rap1 inhibition in diet-induced hyperglycemic mice. Thus, we propose that EPAC2-Rap1 signaling in the hypothalamus could serve as a potential molecular target for therapeutic interventions to mitigate diabetic conditions.

## Methods

### Mice and diets.

Mice were used for all experiments. C57BL/6 mice; *ob/ob* mice; SF1-Cre mice, Tg(Nr5a1-cre)7Low; and Ai9 mice, Gt(ROSA)26Sortm9(CAG-tdTomato)Hze were obtained from The Jackson Laboratory. Rap1a^loxp/loxp^/Rap1b^loxp/loxp^ mice were provided by the National Institute of Mental Health, NIH, Bethesda, Maryland, USA (47). Rap1^ΔSF1^ mice were generated using the following breeding strategy: *Rap1a* and *Rap1b* double-floxed male mice were crossed with female SF1-*Cre* (Nr5a1-*Cre*) mice (23). From these matings, we produced mice with deletion of *Rap1a* and *Rap1b* in Cre-expressing neurons (*Rap1a^fl/fl^ Rap1b^fl/fl^ SF1-Cre*: Rap1^ΔSF1^) and control mice with floxed *Rap1a* and *Rap1b* genes (*Rap1a^fl/fl^ Rap1b^fl/fl^*). Rap1^ΔSF1^ (*Rap1a^fl/fl^ Rap1b^fl/fl^ SF1-Cre*) and control (*Rap1a^fl/fl^ Rap1b^fl/fl^*) mice were generated by crossing male *Rap1a^fl/fl^ Rap1b^fl/fl^* mice to female *Rap1a^fl/fl^ Rap1b^fl/fl^ SF1-Cre* mice. This breeding scheme produced 50% mice with deletion of *Rap1a* and *Rap1b* and 50% control mice with floxed *Rap1a* and *Rap1b* genes. To permit the identification of SF1 Cre-expressing neurons in the brain, the SF1-Cre line was mated with a Cre-dependent expression tdTomato reporter line Ai9 mice (61). Rap1^ΔCNS^ mice were produced as described before (42). All mice were maintained on a 12-hour light/12-hour dark cycle condition (lights on 6 am–6 pm) and temperature-controlled environment at 22°C–24°C with ad libitum access to water and normal diet (Pico Lab, LabDiet, 5V5R) or HFD (60% kcal fat; Research Diets, D12492). The care of all animals and procedures conformed to the *Guide for the Care and Use of Laboratory Animals* of the NIH (National Academies Press, 2011) and were approved by the Institutional Animal Care and Use Committee of Baylor College of Medicine (AN-6076).

### Physiological measurements.

Body weight was measured weekly. Blood samples were collected via the saphenous vein from 4-hour-fasted mice without anesthesia. Serum was isolated after centrifugation (5000*g* for 10 minutes) at 4°C and stored at –80°C. Blood glucose levels were determined in freshly withdrawn blood from the tail vein by using a OneTouch Ultra blood glucose meter. Plasma insulin was analyzed with a Milliplex MAP Mouse Metabolic Hormone Magnetic Bead Panel kit (MilliporeSigma). For glucose tolerance tests, food was removed at 5 pm to initiate an 18-hour fast. The following morning at 11 am, mice were injected with d-glucose (1.5 g/kg), and blood glucose was measured at the indicated time periods from tail vein. For insulin tolerance tests, food was removed at 9 am for 4-hour fasting. At 1 pm, insulin (1 U/kg) was injected intraperitoneally, and blood glucose was measured at the indicated periods from the tail vein. Similarly, we performed a glucose tolerance test for ESI-05–treated mice. Intracerebroventricular surgery was carried out on C57BL/6 mice fed an HFD for 21 weeks, Rap1^ΔCNS^ mice, or 12-week-old *ob/ob* mice. One week after the i.c.v. surgery, the mice were injected with vehicle or ESI-05 (0.2 nmol/mouse) at 5 pm, and food was removed at the same time. The following morning at 9 am, the mice were injected again with vehicle or ESI-05. Two hours after the last bolus injection, a glucose tolerance test was performed.

### Body composition and energy expenditure measurements.

Whole-body composition was measured using NMR imaging (EchoMRI). Body weight– and body composition–matched 9-month-old control and Rap1^ΔSF1^ mice fed normal chow were used. Mice were first acclimatized to the metabolic cages and housed individually for 3 days before measurements were taken. Metabolic parameters, including O_2_ consumption, CO_2_ production, respiratory exchange ratio, heat production, ambulatory activity, and food intake, were determined by using the Columbus Instruments Comprehensive Lab Animal Monitoring System.

### Leptin sensitivity test.

Mice were singly housed and acclimatized for 1 week prior to the study. Body weight– and adiposity-matched, normal chow–fed, 24-week-old control and Rap1^ΔSF1^ mice were injected intraperitoneally with vehicle (Dulbecco’s PBS, dPBS; MilliporeSigma, D8537) twice a day (5 pm and 9 am) for 4 consecutive days. Three days after the last vehicle treatment, mice were injected intraperitoneally with leptin (3 mg/kg, Harbor-UCLA Research and Education Institute) twice a day for 4 consecutive days. Food intake and body weight were measured daily. Similarly, we performed a leptin sensitivity test for HFD-fed mice. Control and Rap1^ΔSF1^ mice were placed on an HFD for 20 weeks, and then the mice were injected with vehicle or leptin twice a day. Body weight and food intake were measured daily.

### Cannula implantation, AAV injection, and treatments.

Mice were anesthetized with isoflurane and positioned in a stereotaxic frame. For cannula implantation, the skull was exposed, and a 26-gauge single stainless steel guide cannula (C315GS-5-SPC, Plastics One) was implanted into the third cerebral ventricles (−0.45 mm from bregma, ±0.9 mm lateral, −2.5 mm from the skull). The cannula was secured to the skull with screws and dental cement. After i.c.v. cannulation, the mice were housed singly and given at least 1 week to recover. On experimental days, the mice were infused with 1 μL of each solution: vehicle (dPBS or dimethyl sulfoxide), leptin (2 μg/mouse), ESI-05 (0.2 nmol/mouse, Axxora, BLG-M092-05), or leptin/ESI-05. To produce Rap1^ΔHYP^ mice in which *Rap1a* and *Rap1b* were deleted in the hypothalamus in vivo, *Rap1a* and *Rap1b* double-floxed littermates received stereotaxic injections ofAAV2-CMV-Cre-GFP or AAV2-CMV-GFP (UNC Vector Core) in both sides of the hypothalamus (0.5 μL/side, −1.7 mm from bregma, ± 0.33 mm lateral, −5.7 mm from the skull based on Franklin and Paxinos’ *Mouse Brain Atlas*, ref. 62). To express human Rap1^V12^ in the hypothalamus, AAV-DJ-Rap1^V12^-GFP or AAV-DJ-GFP was injected into the hypothalamus of C57BL/6J mice similarly as described above. The mice were given at least 1 week to recover.

### Hyperinsulinemic-euglycemic clamps.

Hyperinsulinemic-euglycemic clamp studies were performed at the Mouse Metabolism and Phenotyping Core at Baylor College of Medicine as described previously (63, 64). Mice were anesthetized, and a midline neck incision was made to expose the jugular vein. A microcannula was inserted into the jugular vein, threaded into the right atrium, and anchored at the venotomy site. Mice were allowed to recover for 4 days with ad libitum access to water and food. Following an overnight fast, the conscious mice received a primary infusion (10 μCi) and then a constant-rate intravenous infusion (0.1 μCi/min) of chromatography-purified [3-3H]-glucose using a syringe infusion pump. For determination of basal glucose production, blood samples were collected after 50 and 60 minutes of labeled glucose infusion. After 60 minutes, mice were infused continuously for 2 hours with human insulin (2.5 mU/kg/min). Simultaneously, 25% glucose was infused using another infusion pump at a rate adjusted to maintain the blood glucose level at 100–140 mg/dL (euglycemia). Blood glucose concentration was measured every 10 minutes by a glucometer. Glucose production rate, peripheral glucose disposal rate, and GIR were then calculated. To estimate insulin-stimulated glucose uptake in individual tissues, 2-[14C]-deoxyglucose (2DG) was administered as a bolus (10 μCi) at 45 minutes before the end of the clamps. At the end mice were euthanized, and tissues were snap-frozen using liquid nitrogen for tissue-specific glucose uptake. Glucose uptake in different tissues was calculated from plasma 2DG profile fitted with double exponential curve and tissue content of 2-[14C]deoxyglucose-6-phosphate.

### Total protein extraction and Western blot analysis.

Proteins were extracted by homogenizing samples in lysis buffer (25 mM Tris-HCl at pH 7.4, 150 mM NaCl, 1% NP-40, 1 mM EDTA, 5% glycerol) (87787 and 87788 Pierce IP Lysis Buffer, Thermo Fisher Scientific) with protease and phosphatase inhibitor cocktails (1:100, 78442, Thermo Fisher Scientific). The VMH and ARC were collected as follows: after brief anesthetization with isoflurane, mice were decapitated, and the whole brain was removed. Frontal sections of the hypothalamus were prepared using a brain matrix (1 mm thick), and the VMH and ARC were microdissected under a fluorescence stereomicroscope (Nikon, stereozoom SMZ1500), frozen immediately in dry ice, and stored at –80°C. Equal amounts of the samples were separated by SDS-PAGE and transferred to a nitrocellulose membrane by electroblotting. The following primary antibodies were used for Western blot assays: phosphorylated Akt antibody (1:1000, Cell Signaling Technology, 4060), Akt antibody (1:1000, Cell Signaling Technology, 2920), Rap1 antibody (1:1000, Santa Cruz Biotechnology, sc-398755), and antibody against β-actin (1:1000, Cell Signaling Technology, 4970S). After incubation with primary antibodies for 24–72 hours at 4°C, the membranes were incubated with the following secondary antibodies conjugated to a fluorescent entity: IRDye 680RD goat anti-rabbit IgG (LI-COR Biosciences, 926-68071) and/or IRDye 800CW goat anti-mouse IgG (LI-COR Biosciences, 926-32210), with gentle agitation for 1 hour at room temperature. To measure the fluorescence intensity, the Odyssey IR imaging system (LI-COR Biosciences) was used.

### Total RNA extraction and quantitative real-time PCR.

The VMH and ARC samples were collected as described above, and total RNA was isolated using the PicoPure RNA Isolation Kit (Applied Biosystems, Thermo Fisher Scientific). cDNA was generated by iScript RT Supermix (Bio-Rad Laboratories) and used with SsoAdvanced Universal SYBR Green Supermix (Bio-Rad Laboratories) for quantitative real-time PCR analysis. qPCR assays were performed using a CFX384 Touch Real-Time PCR Detection System (Bio-Rad Laboratories). Normalized mRNA levels were expressed in arbitrary units obtained by dividing the averaged, efficiency-corrected values for sample mRNA expression by that for cyclophilin RNA expression for each sample. The resulting values were expressed as fold change above average control levels. The primer sequences were as follows: human Rap1a (F-AAAGCTAGCATGCGTGAGTACAAGCTAGT and R-TCAACCGGTGAGCAGCAGACATGATTTCTTTTTAGG), mouse Rap1b (F-GCATCATGCGTGAGTACAAG and R-ACCTCGACTTGCTTTCTGTAG), mouse Rap1b (F-GTGAATATAAGCTCGTCGTGC and R-ACACTGCTGTGCATCTACTTC), Cre (F-AACGCAGTCTCCCTTGTTATG and R-GTCGAAATCAGTCCGCTCAA), or Cyclophilin (F-TGGAGAGCACCAAGACAGACA and RTGCCGGAGTCGACAATGAT).

### Immunohistochemistry.

Under deep anesthesia, mice were intracardially perfused with saline and 10% formalin. The brains were removed, postfixed in 10% formalin, infiltrated with 20% sucrose, and cut into 25 μm slices. The sections were rinsed 6 times for 5 minutes each in PBS and then for 30 minutes in 0.3% hydrogen peroxide in 0.25% Triton X-100 in PBS (PBT). The sections were then incubated for 48–72 hours with phosphorylated STAT3 antibodies (1:3000, Cell Signaling Technology, 9131), Rap1 antibodies (1:1000, Bioss Antibodies, Bs-1504R), NeuN (1:100, MilliporeSigma, MAB377), and SF1 (1:100; R&D Systems, Bio-Techne; PP-N1665-0C) in 3% normal donkey serum with PBT with 0.02% sodium azide. The sections were reacted with biotinylated secondary antibody against rabbit IgG (1:1000, Vector Laboratories, BA-1000) followed by the avidin-biotin-peroxidase complex kit (1:1000, Vectastain Elite ABC kit; Vector Laboratories). These immunoreactivities were visualized by incubation with 3,3′-diaminobenzidine (MilliporeSigma) or fluorescence-conjugated streptavidin (1:500, Life Technologies, Thermo Fisher Scientific). After dehydration through graded ethanol, the slides were immersed in xylene and coverslipped. Images were analyzed using a bright-field Leica microscope.

### Statistics.

The data are presented as the mean ± SEM. Statistical analyses were performed using GraphPad Prism 9 for a 2-tailed unpaired Student’s *t* test or 1- or 2-way ANOVA followed by post hoc Tukey’s or Bonferroni’s tests. *P* < 0.05 was considered statistically significant.

### Study approval.

All procedures to maintain and use the mice followed protocols reviewed and approved by the Institutional Animal Care and Use Committee of Baylor College of Medicine (AN-6076) (Houston, Texas, USA) and the Animal Research Committee of Kyoto University (R2-52) (Kyoto, Japan).

## Author contributions

MF conceived the study. KK, HYL, YF, and MF designed the experiments. KK, HYL, YF, ABDLPG, and PKS performed the experiments. PC and AM contributed reagents and intellectually assisted with the Rap1^V12^ and Rap1^ΔSF1^ mouse studies. KK, HYL, YF, PKS, YX, KO, and MF analyzed the data and interpreted the results. The majority of the manuscript was written by MF with some help from KK and YF. All authors approved the final version of the manuscript.

## Supplementary Material

Supplemental data

## Version 1 05/11/2021

In-Press Preview

## Version 2 06/08/2021

Electronic publication
