# Supplementary material for: Rap1 in the VMH regulates glucose homeostasis
Source: JCI Insight. 2021 Jun 8;6(11):e142545. doi: 10.1172/jci.insight.142545 (PMC8262364; doi:10.1172/jci.insight.142545)

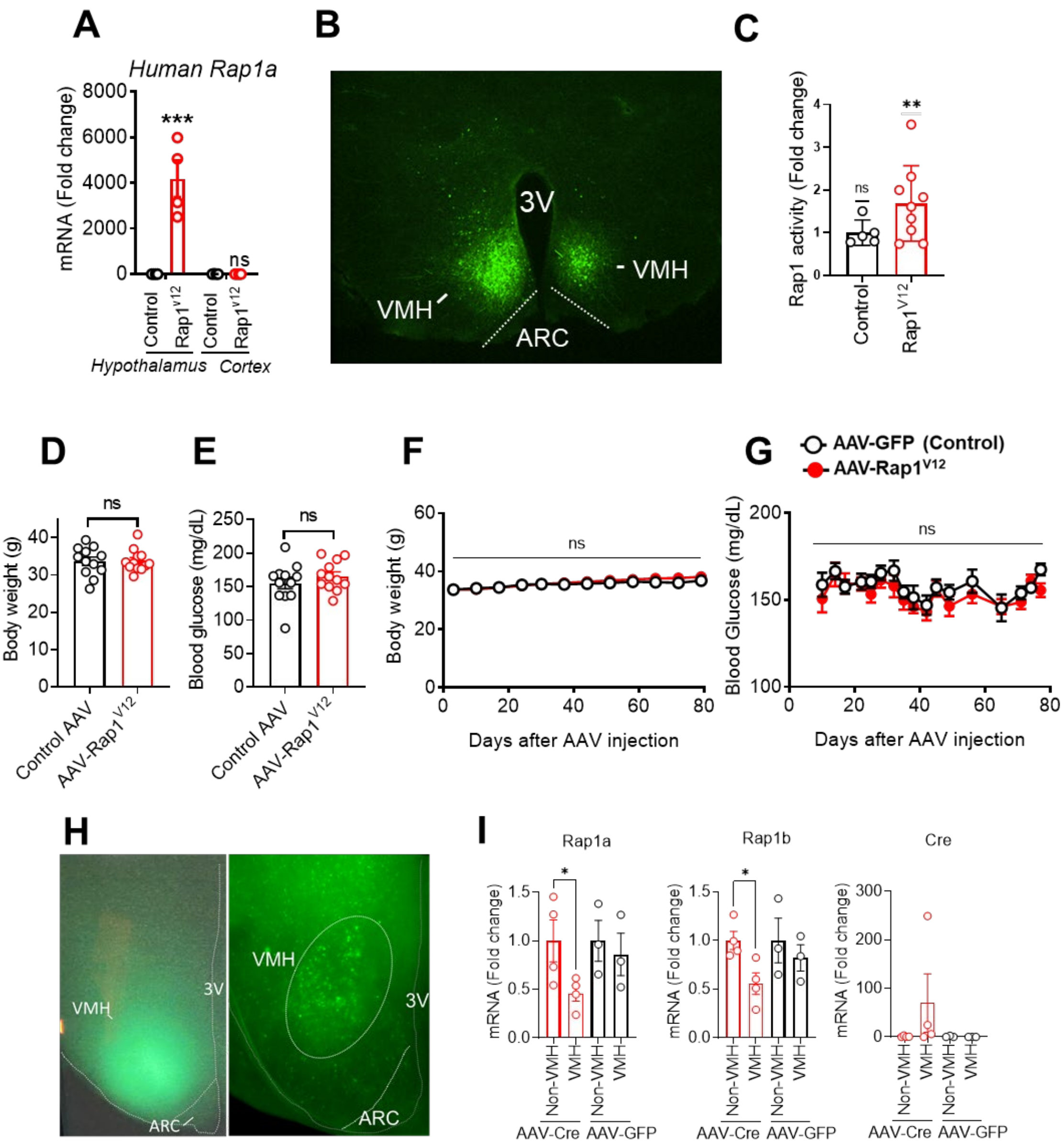

Supplemental Figure 1. Validation of Rap1^{V12} mice and Rap1$^{\triangle HYP}$ mice

(**A**) Relative mRNA levels of human *Rap1a* in hypothalamus and cortex of AAV-Rap1^{v12} or AAV-GFP (control) mice measured by real-time RT-qPCR. (n=4–5). (**B**) Direct visualization of AAV-GFP-Rap1^{V12} in the hypothalamus. (**C**) Total activity of Rap1 was increased in the hypothalamus of mice receiving AAV-Rap1^{V12}. RAP1 activity was measured as previously described (1). (**D** and **E**) Shown are body weight (**D**) and blood glucose (**E**) in AAV-Rap1^{v12} and AAV-Cre mice before HFD feeding was initiated (n = 12-13). (**F** and **G**) Body weight and glucose phenotype of Rap1^{V12} mice under normal chow condition. Shown are body weight (**F**) and blood glucose (**G**) (n = 12-13). (**H**) Direct visualization of AAV-Cre-GFP in the hypothalamus. Shown are a representative low powered stereo-microscopic image (*left*) and a fluorescence microscope image (10 × magnification, *right*) of AAV-Cre-GFP in the hypothalamus. (**I**) *Rap1a* and *Rap1b* mRNA were reduced in the VMH of Rap1$^{\triangle HYP}$, measured by RT-qPCR. *P <0.05, **p <0.01, and ***p <0.001 for t-test. Arcuate nucleus (ARC), ventromedial hypothalamus (VMH). The 3rd ventricle (3V). All error bars are SEM.

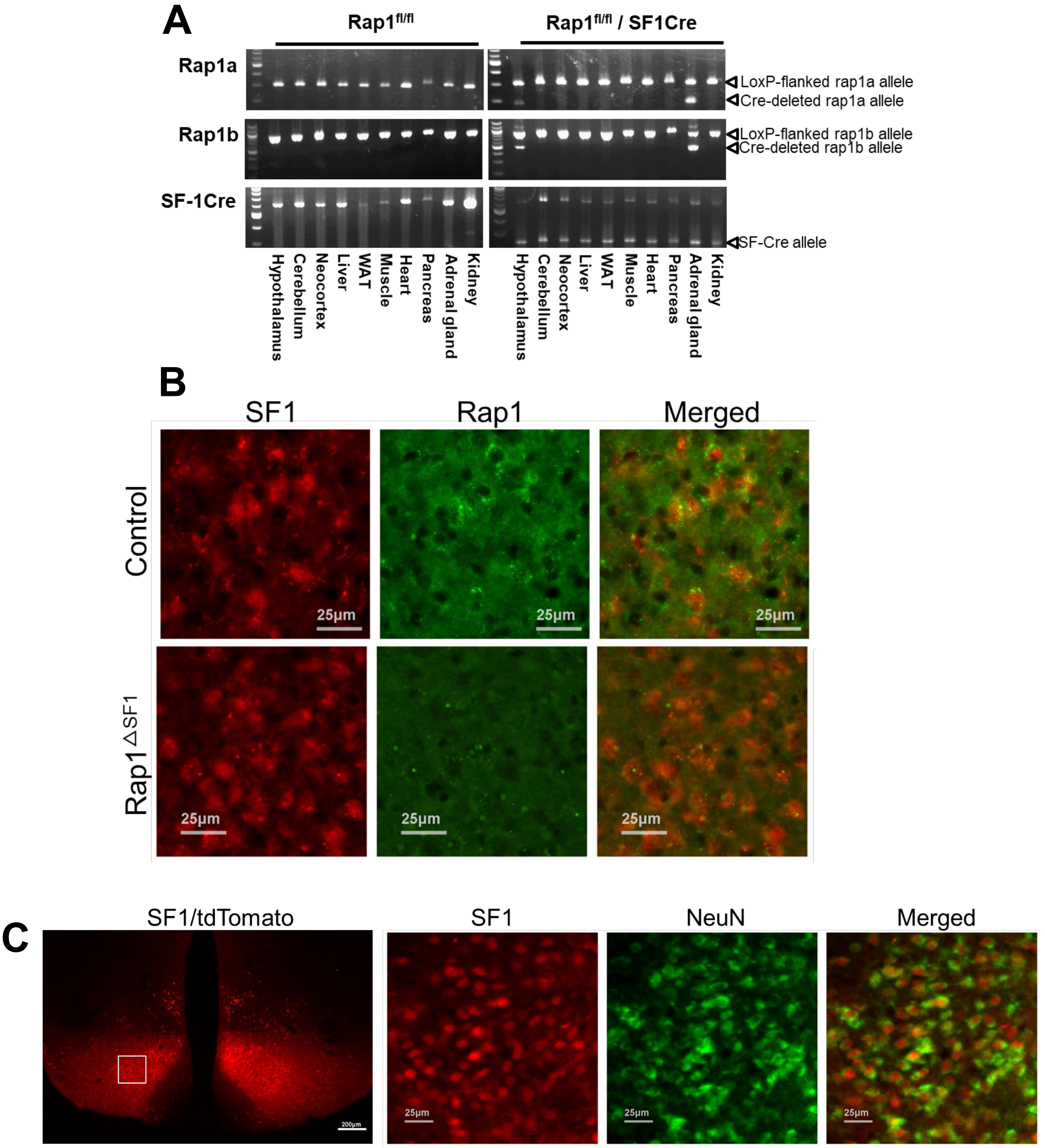

Supplemental Figure 2. Validation of Rap1^{SF1} mice

(**A**) PCR genotyping analyses were performed from several tissues of Rap1$^{\Delta SF1}$ and control mice. Cre-deleted alleles are detected only in hypothalamus and adrenal gland. (**B**) Immunohistochemistry for Rap1 and SF1 to confirm SF1-Cre-mediated ablation of Rap1 in the VMH SF1 neurons in Rap1$^{\Delta SF1}$ mice. (**C**) SF1 positive cells in the VMH are stained with a neural marker NeuN. Anti-NeuN immunofluorescence (a neural marker) was performed on coronal brain sections of SF1Cre/tdTomato mice that produce tdTomato fluorescence in SF1 positive cells. Note that both cytoplasmic and nuclear staining of NeuN was observed in the VMH, which is consistent with the previous studies reporting cytoplasmic NeuN staining in some areas in the brain including the VMH (1) ((1)Daniela Lind Rahul Agrawal, JCI insight).

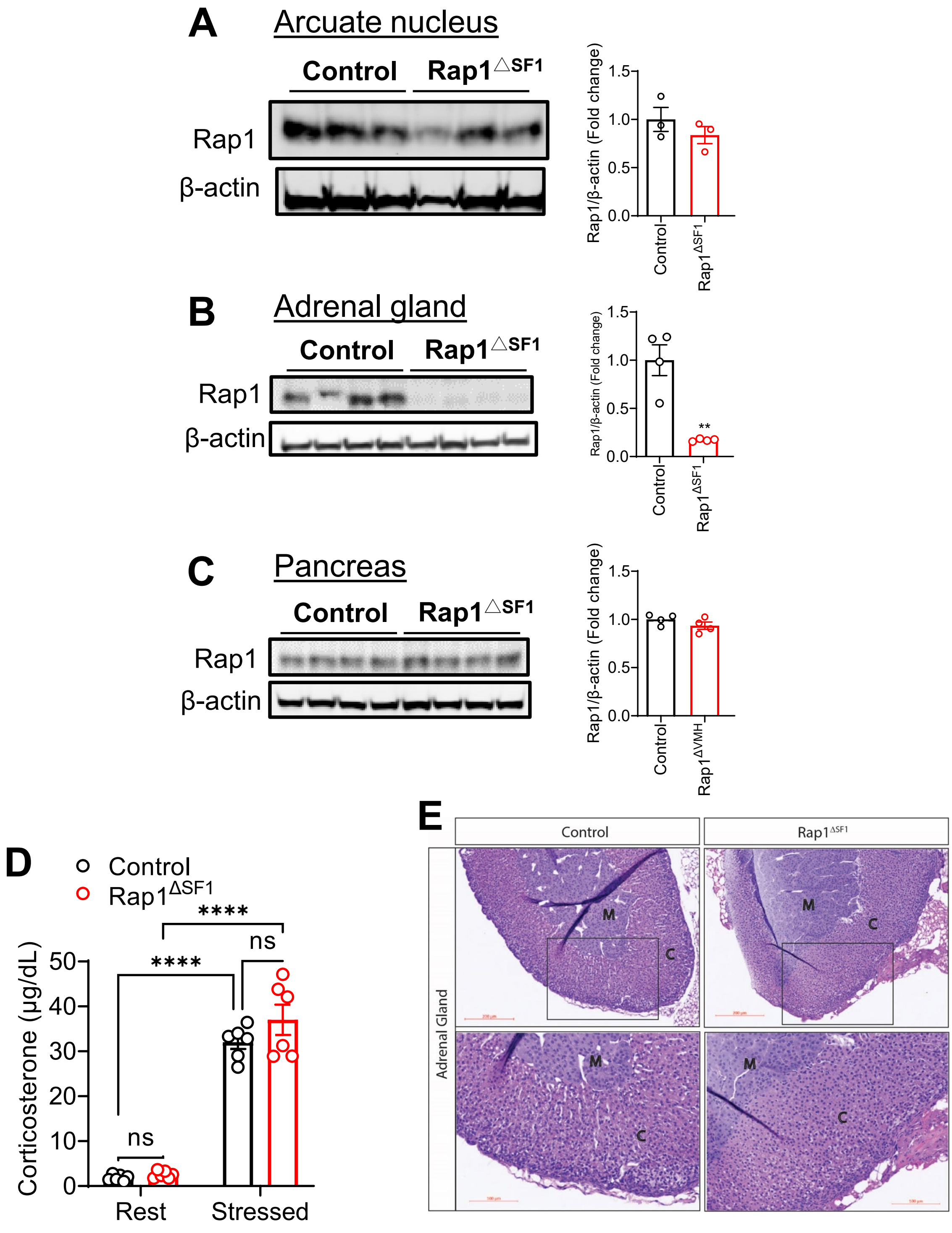

Supplemental Figure 3. Serum insulin levels in Rap1$^{\Delta VMH}$ mice

(**A-C**) Western blot (left) and quantification (right) of Rap1 protein normalized to β-actin in the arcuate nucleus (n = 3, **A**), adrenal gland (n = 4, **B**), and pancreas (n = 4, **C**) in Rap1$^{\Delta SF1}$ and control mice. (**D**) Basal serum corticosterone (Rest) and serum corticosterone after acute stress (Stressed, 15 min of restraint) in Rap1$^{\Delta SF1}$ and control mice (n = 6). (**E**) Representative images of hematoxylin and eosin stained adrenal glands of Rap1$^{\Delta SF1}$ and control mice. **p <0.01 and ****p <0.0001 for t-test in (**B**) or two-way ANOVA followed by Tukey's multiple comparisons test in (**D**). All error bars are SEM.

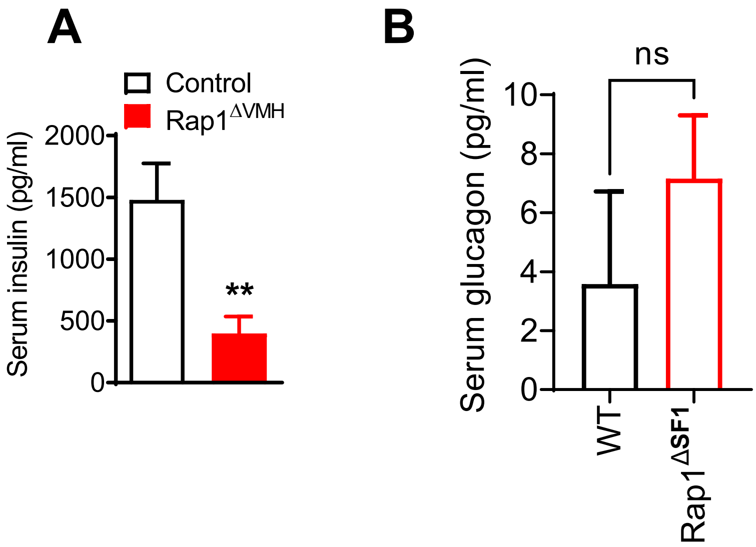

Supplemental Figure 4. Serum insulin and glucagon levels in Rap1$^{\Delta SF1}$ mice
Serum concentrations of insulin (n = 9-15) and glucagon (n = 4-5) in Rap1$^{\Delta SF1}$ and control mice. **P <0.01 for t-test. All error bars are SEM.

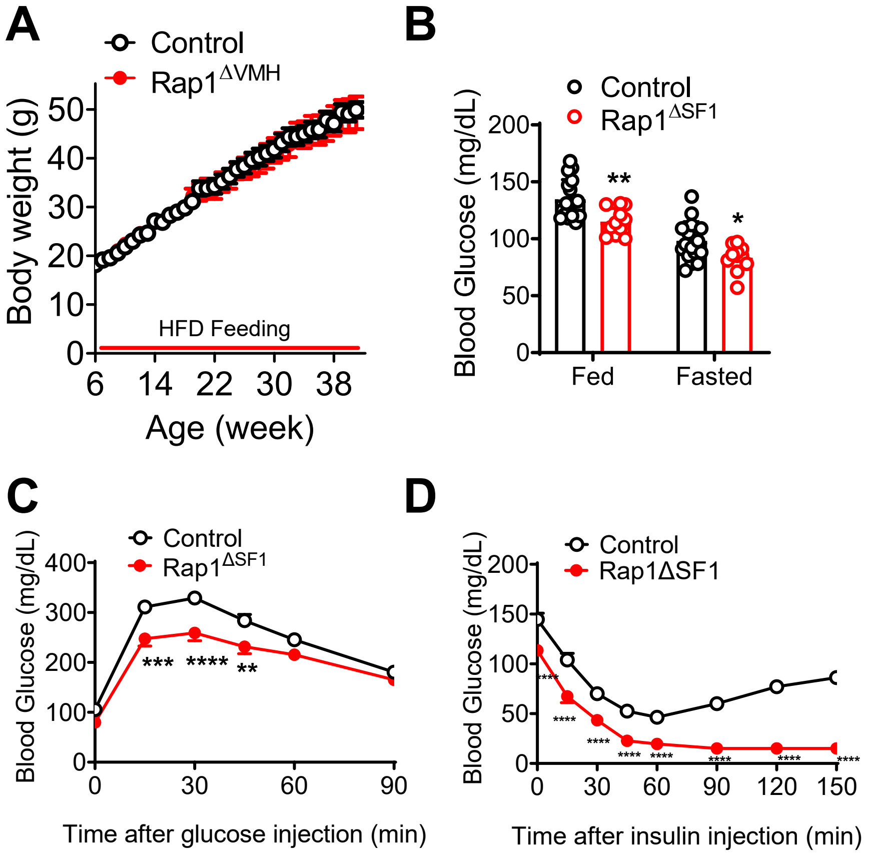

Supplemental Figure 5. Body weight and glucose profiles of Rap1$^{\Delta SF1}$ and control female mice under HFD conditions

(**A**) Body weight in Rap1$^{\Delta SF1}$ female mice (n=8) and their control female mice (n = 16) fed on a HFD starting at 4 weeks of age. (**B-D**) Glucose profiles of HFD-fed Rap1$^{\Delta SF1}$ or control mice (16 weeks of HFD feeding). Glucose (**B,** n = 11-17), GTT (**C,** n = 8) and ITT (**D**, n = 8-9). *P<0.05, **P<0.01, and ***P<0.001 for t-tests in (**B**) or two-way ANOVA followed by Bonferroni's multiple comparisons tests in (**A**, **C** and **D**). All error bars are SEM.

Supplemental Figure 5

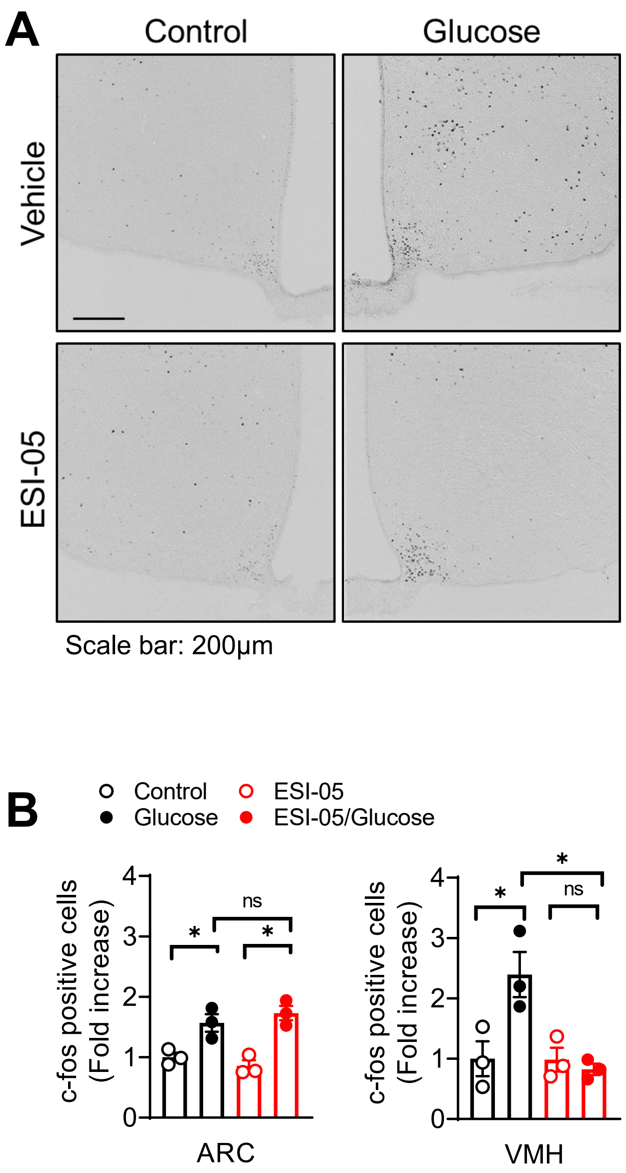

Supplemental Figure 6. ESI-05 inhibits ICV glucose-induced cFos induction in the VMH but not in the ARC. (**A** and **B**) C57BL/6J mice (n = 3) were i.c.v. administered ESI-05 (0.5 nmol, twice) or vehicle followed by i.c.v. glucose (100 μg) 2 hours after the last ESI-05 injection. Ninety minutes later, brain was perfused, sliced and subjected to c-Fos immunohistochemistry. Representative images (**A**) and quantification of c-Fos staining in the ARC and the VMH (**B**). *p<0.05 by 2-way ANOVA followed by Tukey's multiple comparisons test.

Full unedited gels for Figure 1B

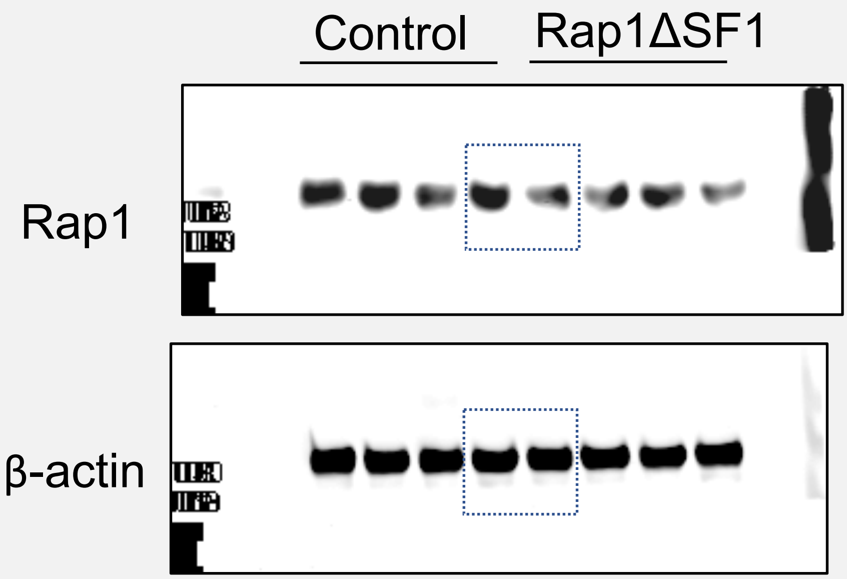

Full unedited gels for Supplemental Figure 3D

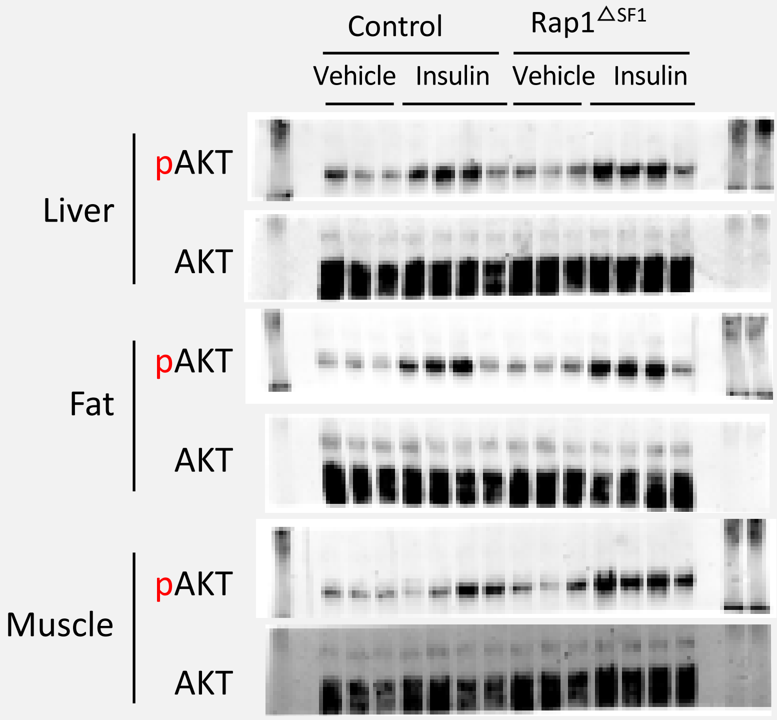

Supplement: Supplemental data [file jciinsight-6-142545-s114.pdf]
